# PPAR**γ** and Proline Oxidase in Cancer

**DOI:** 10.1155/2008/542694

**Published:** 2008-07-28

**Authors:** James M. Phang, Jui Pandhare, Olga Zabirnyk, Yongmin Liu

**Affiliations:** ^1^Metabolism and Cancer Susceptibility Section, Laboratory of Comparative Carcinogenesis, Center for Cancer Research, National Cancer Institute, Frederick, MD 21702-1201, USA; ^2^Basic Research Program, SAIC-Frederick, National Cancer Institute, Frederick, MD 21702-1201, USA

## Abstract

Proline is metabolized by its own specialized enzymes with their own tissue and subcellular localizations and mechanisms of regulation. The central enzyme in this metabolic system is proline oxidase, a flavin adenine dinucleotide-containing enzyme which is tightly bound to mitochondrial inner membranes. The electrons from proline can be used to generate ATP or can directly reduce oxygen to form superoxide. Although proline may be derived from the diet and biosynthesized endogenously, an important source in the microenvironment is from degradation of extracellular matrix by matrix metalloproteinases. Previous studies showed that proline oxidase is a p53-induced gene and its overexpression can initiate proline-dependent apoptosis by both intrinsic and extrinsic pathways. Another important factor regulating proline oxidase is peroxisome proliferator activated receptor gamma (PPAR*γ*). Importantly, in several cancer cells, proline oxidase may be an important mediator of the PPAR*γ*-stimulated generation of ROS and induction of apoptosis. Knockdown of proline oxidase expression by antisense RNA markedly decreased these PPAR*γ*-stimulated effects. These findings suggest an important role in the proposed antitumor effects of PPAR*γ*. Moreover, it is possible that proline oxidase may contribute to the other metabolic effects of PPAR*γ*.

## 1. INTRODUCTION

PPAR*γ* can regulate inflammatory responses to prevent chronic inflammation [1], but more importantly, it plays an important role in the sensing and regulation of metabolism [2]. These functions, especially the regulation of metabolism, may be involved in the documented ability of PPAR*γ* to modulate the malignant phenotype [3]. This aspect of PPAR*γ* articulates with the resurgence of interest in metabolism and cancer [4, 5] which has underscored the 50-year old findings of Warburg that the metabolism of tumor cells is deranged; aerobic glycolysis rather than oxidative phosphorylation is the mode of tumor metabolism [6]. Recent findings suggest that many oncogenes and suppressor proteins target metabolic pathways, and in the context of Warburg's early discovery, they form a new, revealing paradigm [7]. The survival and malignant potential of a tumor are critically dependent on its adaptation to a variety of stress situations and nutrient limitations. To generate adequate energy from the relatively inefficient glycolytic pathway, the flux from glucose to lactate must be maintained at a high rate [8]. Thus, vascularity and neoangiogenesis as a response not only to hypoxia but also to the depletion of nutrients play a critical role in tumor progression [9]. In this context, the mobilization of proline from the degradation of extracellular matrix in the tumor microenvironment has come to our attention. The use of proline as alternative stress substrate and the regulation of this response by stress signals has been a focus of our research effort.

## 2. PROLINE METABOLISM

**Figure 1:**
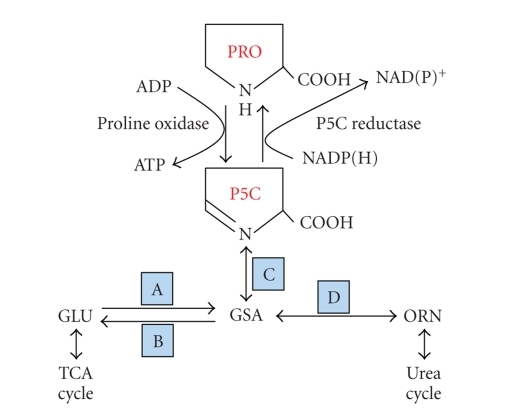
Proline metabolic pathway. Abbreviations: PRO, proline; P5C, Δ^1^-pyrroline-5-carboxylate; GLU, glutamate; GSA, glutamic-gamma-semialdehyde; ORN, ornithine. Enzyme names not shown: A, P5C Synthase; B, P5C dehydrogenase; C, spontaneous; D, ornithine aminotransferase.

Proline is the only secondary amino acid incorporated into protein. Because the alpha nitrogen is contained within a pyrrolidine ring, proline cannot be metabolized by generic amino acid enzymes, that is, aminotransferases, decarboxylases, and racemases [10, 11]. Instead, a special family of enzymes evolved with their own subcellular localizations and mechanisms of regulation. There is a little overlap between the activity of these enzymes and that for generic amino acids. Thus, the metabolic system is distinct and can be responsive to special metabolic requirements. The enzymes for the proline metabolic scheme had been characterized by the 1960s and the general system is shown schematically in Figure 1. Pyrroline-5-carboxylate, in tautomeric equilibrium with glutamic-*γ*-semialdehyde, is a central intermediate. It is not only the committed precursor of proline but also the immediate product of proline degradation. Importantly, it is an obligate intermediate bridging the urea cycle and the tricarboxylic acid cycle and can play an anaplerotic role for both metabolic cycles [10, 11]. The complete metabolic system is not present in all tissues.

The role of proline in proteins has been characterized and reviewed by others [12], and the topic is outside the scope of this review. However, functions beyond its contribution to proteins have also been recognized in a variety of animal and plant species. In prokaryotes, proline is thought to have antioxidant and osmoprotective functions [11]. Regulatory roles have been proposed for parasitic trematodes although the mechanisms are not understood [13]. In a variety of higher plants, proline is thought to be an osmoprotectant and the metabolism of proline has been linked to the synthesis of polyphenolic compounds [14]. Proline has been identified as a critical metabolic substrate in the initiation of flight in insects. In addition, insects can detect and are attracted to proline. The finding that proline is at high concentrations in plant floral nectar has led to the proposal that proline is the basis of a coevolution to optimize insect-mediated pollination [15]. During the molecular biological explosion of the 1990s, the genes for proline metabolism were cloned from a variety of sources, making possible studies defining functions for this special metabolic system.

An interesting feature of proline metabolism is that the interconversions of proline and pyrroline-5-carboxylate form a *proline cycle*. Proline oxidase (POX), a.k.a. proline dehydrogenase (PRODH), is tightly bound to mitochondrial inner membranes (the enzyme will be designated POX, but the gene will be referred to as *PRODH*). The enzyme is a flavoprotein and electrons from proline are passed into the electron transport chain at site II with cytochrome c as the electron acceptor [10, 11]. Pyrroline-5-carboxylate, the product of proline degradation, can be converted to glutamate and *α*-ketoglutarate to contribute anaplerotically to the TCA cycle [11]. However, it is also converted back to proline by pyrroline-5-carboxylate reductase in the cytosol to form a metabolic cycle. Coupled by pyridine nucleotides (NADP/NADPH preferentially over NAD/NADH), the proline cycle forms a metabolic interlock with glucose-6-phosphate dehydrogenase and the pentose phosphate pathway and serves as a redox shuttle to convert reducing potential from the pentose phosphate pathway into an ATP-generating system in mitochondria [16–18]. The magnitude of ATP generation, however, is small compared to the TCA cycle and oxidative phosphorylation. The glycolytic pathway, with optimized flux, also can generate ATP more efficiently. Thus, the contribution of the proline cycle to redox and energetics was considered trivial previously. However, as the mechanisms for upregulating POX were elucidated, it became clear that the system serves as an important accessory source for energy under stress conditions.

Proline is available from dietary proteins and can be biosynthesized from either glutamate or ornithine [10, 11]. However, an abundant source is from degradation of collagen in the extracellular matrix, connective tissue, and bone [19]. Since 25% of the residues in collagen is either proline or hydroxyproline and collagen is the most abundant (by mass) protein in the body, it serves as an ample reservoir of proline. Additionally, matrix metalloproteinases (MMPs), the family of enzymes which degrade collagen and other proteins in the extracellular matrix, are markedly upregulated under a variety of conditions. Importantly, upregulation of MMPs occurs during tumor progression and invasion [20, 21] as well as during inflammation and wound healing [22, 23]. MMP upregulation has been considered an important physical component of invasion, that is allowing for tumor cells to escape from their basement membrane site and migrate through tissue. Recently, it has been shown that a variety of biologically active factors are released from binding sites on ECM with activation of the MMPs [24]. However, the utilization of proline or hydroxyproline as a source of metabolic substrate has not been considered. That degradation of collagen occurs during carcinogenesis in the skin tumor model has been convincingly demonstrated [25]. Recently, using breast and prostate cancer xenografts and novel imaging methodology, investigators have shown that hypoxia mediates collagen fiber breakdown and restructuring [26].

## 3. POX AND APOPTOSIS

**Figure 2:**
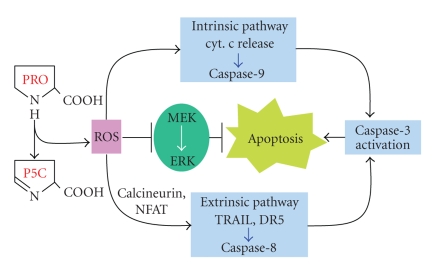
Proline oxidase-induced apoptosis. Abbreviations: ROS, reactive oxygen species; TRAIL, tumor necrosis factor related apoptosis-inducing ligand; DR5, death receptor 5 NFAT, nuclear factor of activated T cells; MEK, MAP kinase; ERK, extracellular-signal regulated kinase.

P53 is considered the most important cancer suppressor protein [27]. It is mutated in 85% of all human tumors and germ-line mutations in p53 result in the Li-Fraumeni syndrome, a familial syndrome with predisposition to early cancers in a variety of tissues [28]. To screen for p53 target genes, Polyak et al. [29] used an adenoviral-p53 expression construct and serial analysis of gene expression. Only 14 out of 7202 genes monitored were induced more than 7-fold, and POX was one of these and designated as p53-induced gene-6 (PIG6). Using a construct where POX expression was under the control of tetracycline, the overexpression of POX produced proline-dependent ROS [30] and induced proline-dependent apoptosis [31–35]. Subsequently, it was shown that POX overexpression produced its effects through generation of proline-dependent mitochondrial superoxide (Figure 2) [34]. It is this superoxide which plays a critical role in signaling to produce not only the release of cytochrome c from mitochondria and the activation of the caspases in the intrinsic (mitochondrial) limb of programmed cell death, but also it activated the extrinsic (death receptor) limb by increasing the production of TRAIL [35]. A number of other signaling systems respond to POX-mediated signaling including downregulation of MEK/ERK phosphorylation [35], downregulation of COX-2 with decreased PGE2 production, and blockade of the progression through the cell cycle [36].

The findings from the tissue culture system have been translated into an animal model. In studies using DLD-POX cells to form xenografts in athymic mice, the expression of POX markedly inhibited tumor formation [37]. In mice given doxycycline to suppress POX expression in DLD-POX cells, or in animals injected with DLD-vector cells, tumors formed rapidly. By week 2 all these animals developed palpable tumors and by week 3, the animals had to be sacrificed due to the size of the tumors. By contrast, in mice without doxycycline in which POX was overexpressed, few tumors were detected. By week 2, only 1 out of 16 animals had palpable tumors. Thus, the expression of POX markedly inhibited the formation of xenografts.

The relevance of these changes in POX was pursued by immunohistochemical studies in human tissues. Ninety-two paired normal and cancer tissues from a variety of tumors were examined using immunohistochemistry. The findings were striking from gastrointestinal tumors (stomach, colon, pancreas) in which the level of POX expression was markedly decreased or undetectable in 79% of the tumors [36]. We are currently investigating the genetic or epigenetic mechanism for the decrease in POX expression, but based on these findings, we propose that POX is a potential cancer suppressor protein.

**Figure 3:**
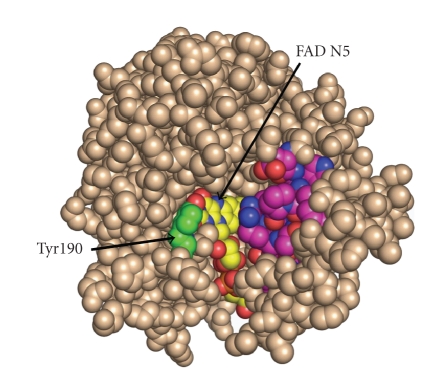
Structure of proline dehydrogenase (proline oxidase) from *Thermus thermophilus*. The flavine adenine dinucleotide at the active site is shown in yellow. The flexible alpha helix adjacent to the FAD is shown in violet and blue. Access of the FAD to solvent O_2_ allows direct reduction of O_2_ to form superoxide radicals. The figure is used with permission from Dr. Jack Tanner, University of Missouri-Columbia, and the Journal of Biological Chemistry.

The mechanism for the POX-mediated, proline-dependent generation of superoxide may be due to leakage of electrons from the electron transport chain, a mechanism proposed for other sources of mitochondrial superoxide. However, recent studies from structural biology suggest that the generation of superoxide is an intrinsic property of the enzyme. White et al. [38] described interesting findings using recombinant *Thermus thermophilus* POX/PRODH. Unlike the POX/PRODH from certain prokaryotic species, for example *Escherichia coli*, which have a bifunctional enzyme, embodying the activities of POX and pyrroline-5-carboxylate dehydrogenase in a single protein, the enzyme from *T. thermophilus* is monofunctional and produces pyrroline-5-carboxylate in a manner similar to the enzyme in animal tissues, and thus may serve as a good model for human POX [38]. These workers found that the flavin adenine dinucleotide is located in a domain exposed to solvent oxygen. Thus, the electrons from proline can be used to reduce oxygen to superoxide (Figure 3). In addition, they found an adjacent *α*-helix which can shield the FAD and block its access to solvent oxygen. The interpretation of these findings includes the intriguing possibility that POX can be switched from an ATP-generating function to a superoxide-producing function. Although a number of enzymes have been proposed as generators of superoxide, these enzymes are cytosolic (xanthine oxidase) or are associated with cell membranes (NADPH oxidase) with their own specified functions.

These aforementioned functions of POX have been emphasized for their relevance to cancer, but another function deserves mention. Proline functions as a neurotransmitter, inhibiting glutamatergic neurons [39]. Additionally, a high-affinity transporter has been discovered and cloned from the brain [39]. The relevance to neurological systems extends to lower species. Mutations in POX/PRODH result in “sluggishness” in *Drosophila melanogaster* [40] and the PRO/Re mice, defective in POX/PRODH, exhibit “gating” defects, a functional neurologic defect [41]. In humans, mutations in *PRODH* have been associated with risk for early schizophrenia [42]. Although there has been a number of studies supporting or contradicting this conclusion, evidence supports the relevance of POX mutations. It has been shown that the mutations in PRODH associated with the neuropsychiatric syndrome have a biochemical phenotype with markedly decreased activity in the enzyme [43].

## 4. REGULATION OF POX

The induction of POX by p53 suggested that it served special functions and was not simply a “housekeeping enzyme.” To screen for potential regulators, Pandhare et al. [44] made a POX-promoter, luciferase-reporter construct, and cotransfected a variety of transcriptional factors corresponding to binding sites identified in the *PRODH* promoter. Although Jun, Fos, and p65 of NF-*κ*B produced modest stimulatory effects (<2-fold), a marked activation of the *PRODH* promoter was observed with cotransfection of PPAR*γ*. This finding was interesting, indeed, since this pleiotropic factor not only plays an important role in metabolism [2], especially of adipocytes, but also it is an important modulator of inflammatory responses [1]. The wide use of the thiazolidinediones (TZDs) in the management of hyperglycemia in type 2 diabetes mellitus is an example of the former [45]. For the latter, some investigators have suggested that PPAR*γ* provides a mechanism to downregulate inflammatory stress responses and avoid the pathologic consequences of chronic inflammation [46]. Attracting considerable attention recently is the finding in a variety of cultured cancer cells that TZDs will block cell proliferation and induce apoptosis [47–49]. Epidemiologic data from patients with type 2 DM treated with TZDs suggest that these ligands of PPAR*γ* are protective against lung cancer but not against colon or prostate cancer [50]. With the impressive in vitro data and suggestive findings from epidemiology, oncologists have proposed that PPAR*γ* is an attractive target for cancer treatment.

## 5. MECHANISM OF TZDs IN INDUCING *PRODH*

**Figure 4:**
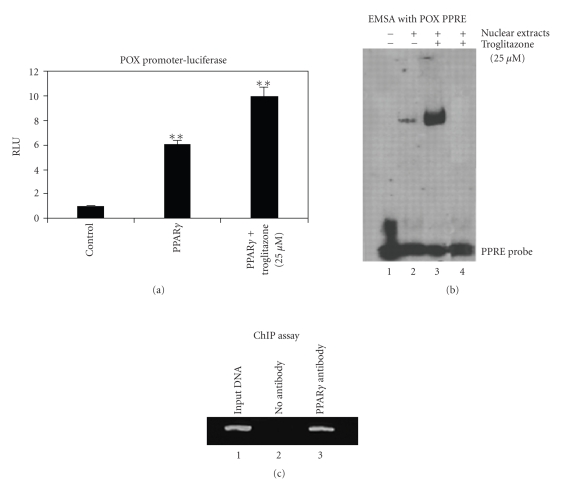
Induction of proline oxidase by PPAR*γ* and its pharmacologic ligand, troglitazone. (a) Activation of the POX promoter using a luciferase reporter assay. HEK 293 colorectal cancer cells were transfected with equivalent amounts of cDNA of PPAR*γ* or vector plasmid as control. The cells were also transfected with *POX*-Luc and pRL-null. Troglitazone (25 *μ*M) or Me_2_SO in control was added after 10 hours as indicated. At 24–36 hours after transfection, the cell lysates were harvested, and the *POX* promoter luciferase activity was determined using the Dual Luciferase Assay kit. (b) Troglitazone increases the binding of PPAR*γ* to the PPRE in the *POX* promoter. HCT 116 colorectal cancer cells were treated with or without 25 *μ*M troglitazone for 36 hours and nuclear extracts were prepared. The binding of PPAR*γ* to the PPRE was evaluated by an electrophoretic mobility shift analysis assay using the double-stranded POX-PPRE oligonucleotide probe. Unlabeled POX-PPRE probe (100x) was used as a competitor (lane 4). (c) Chromatin immunoprecipitation assay of the *POX* promoter in troglitazone-treated HCT 116 cells. HCT 116 cells were incubated with 1% formaldehyde to fix protein-DNA complexes. DNA was sheared by sonication. Soluble chromatin-DNA complexes were immunoprecipitated using PPAR*γ* antibody and immunoprecipitates were analyzed by PCR with specific primers for the *POX* promoter region containing the POX-PPRE.

Pandhare et al. [44] showed that cotransfection of PPAR*γ* activated the *PRODH* promoter 8-fold, and troglitazone, a widely used TZD before it was taken off the market because of side effects, further increased the magnitude of this activation. The combination of PPAR*γ* expression and troglitazone treatment activated the *PRODH* promoter more than 10-fold (Figure 4). The effect could be generalized to a variety of colorectal cancer cells and could be elicited by four different TZDs. That troglitazone induced POX through a PPAR*γ* mediated binding to the peroxisomal proliferator response element was shown using several methods. First, an electrophoretic shift mobility assay showed a troglitazone-stimulated formation of a nuclear complex with the labeled PPRE sequence from the *PRODH* promoter. That PPAR*γ* was present in this complex was shown with chromatin immunoprecipitation assays. In this assay, formaldehyde was used to cross-link DNA-protein complexes and then the DNA was sheared by sonication. After immunoprecipitation with specific anti-PPAR*γ* antibody, the PPRE sequences of the *PRODH* promoter were amplified using polymerase chain reaction.

Although these studies showed that PPAR*γ* and its pharmacologic ligands are directly involved in the activation of the *PRODH* promoter, the integration of signaling by the PPAR*γ* assembly to physiologically regulate *PRODH* expression may be more complex. The interaction with retinoid-X receptors (RXR) is a requisite for PPAR*γ* function [51]. Moreover, a number of coactivators interact with liganded PPAR*γ* and RXR to form an active transcriptional complex. These include steroid receptor PPAR*γ*-coactivator-1 (PGC-1) and steroid receptor coactivator-1 (SRC-1) [52]. The specific coactivator may depend on the cell type and stimuli. In the context of metabolism, PGC-1 may be especially relevant since it responds to signaling from other metabolism-regulating hormones and cytokines [53]. The specific effect of these coactivators on *PRODH* expression, however, has not been elucidated, but it is an area of emphasis of our current work.

## 6. CONTRIBUTION OF POX TO THE PPAR*γ* EFFECTS ON ROS AND APOPTOSIS

The discovery that PPAR***γ*** has a marked inhibitory effect on cultured cancer cells stimulated a large number of studies using a variety of cancer cells. The TZDs augmented differentiation, slowed proliferation, and induced apoptosis. Although this effect was generally observed, there were a few reports of TZDs actually stimulating the growth of certain cultured cancer cells [54]. Nevertheless, the preponderance of studies showed that TZDs inhibited growth [47–49]. Although the mechanism of this effect was not well understood, several investigators found that TZDs induced the generation of ROS, and they concluded that ROS was the mechanism for inducing apoptosis as has been reported for many experimental models. The actual mechanism by which ROS production was induced by TZDs, however, remained unknown.

Since POX is a p53-induced gene and has been established as a mechanism for generating superoxide that initiates apoptosis, the PPAR***γ*** induction of POX raised the attractive hypothesis that POX may be involved in the apoptotic mechanism observed with the TZDs. To answer this question, Pandhare et al. [44] showed that in colorectal cancer cells, troglitazone not only induced POX, but also markedly increased the production of ROS as has been shown by others in other cultured cells. More importantly, the knockdown of POX with antisense RNA markedly decreased the generation of troglitazone-stimulated ROS. These studies strongly suggested that the ROS presumed to be the mechanism for TZD-stimualted apoptosis was due, at least in part, to its induction of POX. Thus, POX plays an important role in the apoptotic effect of TZDs, at least in tissue culture. This finding was soon confirmed by others. Working with nonsmall cell lung cancer cells, Kim et al. [55] showed that rosiglitazone induced apoptosis through an ROS-dependent mechanism, and that the induction of POX by rosiglitazone played a critical role in the production of apoptosis. These are exciting findings but require further corroboration and extension to other cultured cancer cells.

The effects of TZDs in cultured cells have been extended to several tumor models in animals and the results are encouraging. In athymic mice, the growth rates of xenografts of ovarian, thyroid, and bladder cancer are markedly affected by a variety of PPAR*γ*-stimulating agents [47–49]. Not only is tumor growth inhibited but survival of the host animal is prolonged. Although the mechanism underlying these effects remains unclear, it appears that the cells in the tumors are apoptotic perhaps due to decreased expression of COX-2 [56]. Recent work in our laboratory links POX expression to downregulation of COX-2 [36]. There are direct effects on the tumor as well as effects on angiogenesis. There are no studies of the effects of PPAR*γ* on POX expression in animals or on the role of POX in mediating the PPAR*γ*-mediated antitumor effects.

## 7. PARADOXES AND POSSIBLE SOLUTIONS

The enthusiasm generated by these antitumor effects of PPAR*γ* and the TZDs was somewhat blunted by the finding that in C57Bl/6J-APC^Min/+^ mice, activation of PPAR*γ*-mediated signaling promotes rather than inhibits the development of colon tumors [57]. APC is the tumor suppressor protein in adenomatous polyposis coli and is an integral part of the Wnt/*β*-catenin signaling system. The Min mutation blocks the formation of the tetrameric complex (APC, axin, GSK-3*β*, *β*-catenin) which allows for phosphorylation of *β*-catenin leading to its proteasomal degradation. Accumulated *β*-catenin translocates into the nucleus to form transcriptional complexes with TCF/LEF to induce target genes involved in proliferation [58]. However, in keeping with the earlier reports that activation of PPAR*γ* or its ligands had antitumor effects, recent studies have shown marked reduction in tumor growth or survival of animals with peritoneal carcinomatosis with various PPAR*γ* ligands. These recent studies include ovarian cancers [47], anaplastic thyroid carcinomas [48], and bladder tumors [49]. Thus, the debate continues: “. . . the action of PPAR*γ* on cell cycle, proliferation, differentiation, and apoptosis seems to depend on the cell type and/or the mutational events that predispose tissue to cancer development” [58]. The importance of coactivators or corepressors cannot be overemphasized. Interactions with and contributions of the microenvironment must also be considered in understanding these different effects.

A common target of these signaling pathways is the matrix metalloproteinases (MMP) [59, 60]. Differential effects on these enzymes may explain, in part, the variability in the aforementioned effects of PPAR*γ* activation. Increased PPAR*γ* signaling will downregulate MMP whereas certain MMP are target genes of *β*-catenin/TCF-LEF. The transcriptional system constitutively upregulated by the APC^Min^ mutation increases the expression of MMP-7. Just how these mechanisms articulate for regulating MMP remains unclear. However, in the context of the aforementioned induction of POX by PPAR*γ*, the differential effects on MMP may be relevant. In a given experimental model, the availability of ECM and the effects on MMP may determine the relative availability of proline as a stress substrate for POX. Furthermore, the consequences of POX induction may also be two-edged. Under stimulation of p53, POX can use proline to generate mitochondrial superoxide to initiate apoptosis by both intrinsic and extrinsic pathways [34]. Recent work has shown that POX overexpression will also blockade the cell cycle [61]. Thus, upregulation of POX in the presence of MMP to generate free proline will activate antitumor mechanisms. On the other hand, POX also can generate ATP and it is upregulated by downregulation of mTOR signaling under nutrient stress. With the availability of proline, upregulation of POX can support cell survival [62]. Like several mediators of metabolic regulators, for example, p53 and PPAR*γ*, POX also can play a two-edged regulatory role.

## 8. THE ROLE OF POX IN ANTITUMOR EFFECTS OF PPAR*γ*

Additional work is needed to translate these findings in cultured cancer cells to animal models and eventually to clinical trials. As a first step, studies are being undertaken to monitor the expression of POX in mice administered TZDs. Assuming that certain tissues in intact animals will respond as in cultured cells, the effect of POX upregulation on spontaneous tumors in that tissue can be investigated. The inhibition of POX by proline analogues or the blockade of MMPs, specifically prolidase, may limit the availability of proline in that tissue. Also, control of dietary proline could be important. With the insights gained by these animal studies, it may be possible to design clinical trials in which perturbations of the POX-mediated effects can be pharmacologically attacked as an adjunct to the use of TZDs or other PPAR*γ* activators. Furthermore, PPAR*γ* activation with or without POX can be used in combination with other chemotherapeutic modalities.

## 9. CONTRIBUTION OF POX TO OTHER PPAR*γ*-MEDIATED EFFECTS

The consequences of POX induction and its role in PPAR*γ*-mediated metabolic effects other than that on cancer have not been explored. However, it is intriguing that the well-established metabolic effects of PPAR*γ* could be mediated in part by induction of POX. Nevertheless, the known effects of POX and PPAR*γ* invite speculation, but these specific questions have not been experimentally addressed. Thus, these questions remain in the realm of future plans. Of special consideration are the following effects of PPAR*γ*: (1) increased insulin sensitivity, (2) decreased inflammation, and (3) increased osteopenia.

There are potential links between degradation of proline and insulin-related metabolic effects. Certainly, POX uses proline to generate intermediates for anaplerosis of the TCA cycle which could make oxidative metabolism more efficient. Investigators have cited the importance of these intermediates as building blocks rather than as energy substrates. Furthermore, the metabolic interlock of the proline cycle and glucose metabolism through the pentose phosphate pathway could affect insulin sensitivity since it opens an alternative pathway for glucose metabolism. Thus, glucose would not only be metabolized by oxidative phosphorylation in the TCA cycle and converted to lactate by glycolysis, but also would be converted to CO_2_ by interconversions and cycling through the pentose phosphate shunt.

The PPAR*γ* signaling pathway is frequently considered as a response to inflammatory stress, that is, to prevent chronic inflammation. Inflammatory cells such as macrophages will respond to inflammatory signals such as prostaglandins and this will induce POX in macrophages and induce apoptosis. Furthermore, COX-2 may be regulated by the expression of POX and the generation of proline-mediated ROS [36].

 The final metabolic consideration is the demonstrated effects in animals and in humans that TZDs will result in osteopenia [63]. From histologic and metabolic studies, PPAR*γ* appears to decrease osteogenesis and increase osteoloysis. There are decreased numbers of osteoblasts and increased numbers of osteoclasts [64]. Since bone is primarily made up of calcified collagen, it is not surprising that collagen synthesis is decreased and collagen degradation is increased. Since collagen synthesis requires the incorporation of proline, the degradation of proline by increased POX would be a biochemical process consistent with osteoclastic function.

Another interesting area involves a physiologic/pathophysiologic source of natural ligands for PPAR*γ*, that is, oxidized low-density lipoproteins (oxLDL). Their precursor, low-density lipoproteins (LDL) are synthesized in the liver and are the carriers for 60% of total serum cholesterol, and they are widely known as the “bad cholesterol.” Recent studies suggest that LDL is oxidized in human blood and tissues under various pathological conditions. OxLDL may be an important player in the development of atherosclerosis, promoting apoptosis in endothelial cells, increasing proliferation of smooth muscle cells, and upregulating inflammatory signaling in macrophages. The result is the formation of atheromatous plaques. Mechanisms of oxLDL-induced effects are being intensively investigated, but there is a considerable evidence supporting a role for PPAR*γ* activation [65]. Additionally, oxidized LDL activates p53 [66, 67] and stimulates the formation of mitochondrial ROS [68] to induce cell death. Since all these mechanisms are linked to POX activity, it is tempting to speculate that POX may be involved.

Although oxLDL is mainly associated with atherosclerosis, several studies point to the correlation between serum oxLDL levels and cancer risk in humans [69, 70]. This prompted us to study the possible role of POX in the oxLDL-mediated effects on carcinogenic pathways. First, we transfected breast, prostate, colon, cervical, ovarian, and lung cancer cell lines with the POX promoter-luciferase reporter and found that oxLDL treatment activated the POX promoter in a dose- and time-dependent manner. This effect was further augmented by the addition of 2.5 mM proline. We also found that oxLDL treatment increased POX gene expression as compared to nonoxidized LDL, or a solvent control [Zabirnyk O and Phang JM, unpublished results]. These preliminary studies suggest a role of proline oxidase in the oxLDL-mediated effects on PPAR*γ* activation and initiation of apoptotic cell death.

In summary, POX, a p53-induced gene, is markedly upregulated by overexpression of PPAR*γ* or by the addition of TZDs. The effect is generalizable to a variety of cells and to all the TZDs. The mechanism of this effect appears to be by transcriptional activation by activating the POX promoter at the PPRE site. The PPAR*γ* effect on apoptosis is mediated by the generation of ROS, and knockdown of POX by siRNA markedly decreases or blocks the effects of PPAR*γ* on ROS formation and apoptosis in colorectal cancer cell or nonsmall cell lung cancer cell, respectively. These findings suggest that POX may play a critical role in PPAR*γ*-mediated antitumor effects. Furthermore, it may offer an explanation for the inconsistent findings observed in different animal systems. It also may offer an adjunctive therapeutic approach to optimize the PPAR*γ*-mediated antitumor effects. Finally, a speculative proposal for the articulation of POX-dependent metabolic effects on the metabolic syndrome with PPAR*γ* activation is presented.

## ABBREVIATIONS

NFAT: nuclear factor of activated T-cells
P5C: Δ^1^-pyrroline-5-carboxylic acid
POX: proline oxidase
PPAR*γ*: peroxisome proliferator-activated receptor gamma
PRODH: proline dehydrogenase
ROS: reactive oxygen species
TRAIL: tumor necrosis factor-related apoptosis-inducing ligand
TZDs: thiazolidinediones
